# Adverse Events and Safety Profile of the COVID-19 Vaccines in Adolescents: Safety Monitoring for Adverse Events Using Real-World Data

**DOI:** 10.3390/vaccines10050744

**Published:** 2022-05-09

**Authors:** Chae Won Lee, Soonok Sa, Myunghee Hong, Jihyun Kim, Sung Ryul Shim, Hyun Wook Han

**Affiliations:** 1 Department of Biomedical Informatics, CHA University School of Medicine, CHA University, Seongnam 13488, Korea; leechaewon@chauniv.ac.kr (C.W.L.); soonoksa@chauniv.ac.kr (S.S.); mhhong99486@gmail.com (M.H.); 2 Institute of Basic Medical Sciences, School of Medicine, CHA University, Seongnam 13488, Korea; 3 Institute for Biomedical Informatics, School of Medicine, CHA University, Seongnam 13488, Korea; 4 Department of Pediatrics, Samsung Medical Center, Sungkyunkwan University School of Medicine, Seoul 06351, Korea; jihyun77@skku.edu; 5 Healthcare Big-Data Center, Bundang CHA Hospital, Seongnam 13488, Korea; 6 Department of Health and Medical Informatics, Kyungnam University College of Health Sciences, Changwon 51767, Korea

**Keywords:** COVID-19, vaccines, severe adverse events, BNT162b2, adolescents, safety

## Abstract

A COVID-19 vaccine BNT162b2 (Pfizer-BioNTech) has recently been authorized for adolescents in the US. However, the impact of adverse events on adolescents after vaccination has not been fully investigated. To assess the safety of the COVID-19 vaccine in adolescents, the incidence of adverse events (AEs) in adolescents and adults was compared after vaccination. We included 6304 adolescents (68.14 per 100,000 people) who reported adverse events using vaccine adverse event reporting system (VAERS) data from 10 May 2021 to 30 September 2021. The mean age was 13.6 ± 1.1 years and women (52.7%) outnumbered men. We analyzed severe and common adverse events in response to the COVID-19 vaccine among 6304 adolescents (68.14 per 100,000 people; 52% female; mean age, 13.6 ± 1.1 years). The risk of myocarditis or pericarditis among adolescents was significantly higher in men than in women (OR = 6.61, 95% CI = 4.43 to 9.88; *p* < 0.001), with a higher frequency after the second dose of the vaccine (OR = 8.52, 95% CI = 5.79 to 12.54; *p* < 0.001). In addition, severe adverse events such as multisystem inflammatory syndromes, where the incidence rate per 100,000 people was 0.11 (*n* = 10), and the relative risk was 244.3 (95% CI = 31.27 to 1908.38; *p* < 0.001), were significantly higher in adolescents than in adults. The risk of the inflammatory response to the COVID-19 vaccine, including myocarditis, pericarditis, or multisystem inflammatory syndromes, was significantly higher in men than in women, with a higher frequency in adolescents than in adults. The inflammation-related AEs may require close monitoring and management in adolescents.

## 1. Introduction

The United States Food and Drug Administration (FDA) issued an emergency use authorization for the BNT162b2 vaccine (Pfizer-BioNTech) for use in persons aged ≥ 16 years on 11 December 2020, which was later expanded to include adolescents aged 12–15 years on 10 May 2021 [1]. Recent reports have described myocarditis (inflammation of the myocardium) and pericarditis (inflammation of the pericardium, which is the inner visceral layer that encloses the heart) in young men or adolescents aged 12–18 years following COVID-19 vaccination [2,3].

COVID-19 infection cases were globally [4,5,6,7] reported to account for 1–3% of children and adolescents. Although children typically have relatively mild clinical presentations of SARS-CoV-2 infection with few complications [8], case–fatality rate in children with COVID-19 was reported in 0.3~0.69% [9,10]. Furthermore, multisystem inflammatory syndromes and myocarditis have been rarely reported, and those symptoms are severe [11,12]. In addition, inflammatory disorders such as Kawasaki disease, toxic shock syndrome, secondary hemophagocytic lymphohistiocytosis, and macrophage activation syndrome, have also been reported and are associated with a recent infection with SARS-CoV-2 [13].

Although vaccination provides effective protection against COVID-19 through the rapid development of the COVID-19 pandemic, unexpected adverse events (AEs) resulting from vaccination have not been systematically reported. Severe rare adverse events associated with vaccines may not be identified in phase 3 trials because of the small sample size in the pediatric population, restrictive inclusion criteria, limited duration of follow-up, and trial participants who may differ from the population ultimately receiving vaccines [14]. Safety monitoring of AEs after vaccination is critical to ensure safety, maintain trust, and inform policies. This study aimed to clarify the associated AEs and to understand the spectrum of AEs by incidence rate and risk in adolescents. This study evaluated AEs in order to provide accurate post-vaccination safety information by comparing adolescents and adults.

## 2. Materials and Methods

This study was performed in accordance with guidelines issued by Strengthening the Reporting of Observational Studies in Epidemiology (STROBE) and was approved by the Institutional Review Board of the CHA Bundang Medical Center, CHA University. The ethics board waived the need for individual participation consent because the study involved the analysis of data already collected and the data had individual identifying information removed.

### 2.1. Data

**Figure 1:**
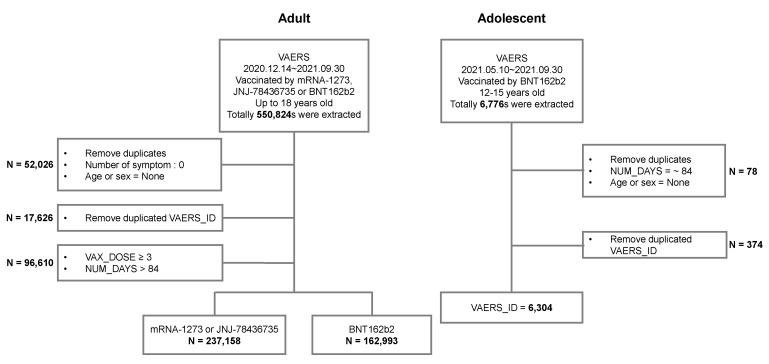
Study workflow.

This retrospective analysis was based on data obtained from VAERS, a post-marketing safety surveillance program, developed in the USA in 1990 by the Centers for Disease Control and Prevention (CDC) and the FDA, which collects information about AEs that occur after the administration of vaccines licensed in the USA [15]. The information obtained is useful as an early warning system for potential safety issues associated with US-licensed vaccines. Vaccine recipients, healthcare providers, and vaccine manufacturers can openly report side effects to VAERS [16]. We used VAERS data from 10 May 2021 to 30 September 2021, to analyze and characterize post-vaccination AEs associated with COVID-19 vaccines authorized for the adolescent population in the USA aged 12–15 years (due to the FDA expanding the emergency use authorization on 10 May). Among all vaccinated individuals, AEs were reported in 6304 adolescents and 162,993 adults, respectively (Figure 1). We used VAERS data from 14 December 2020 to 30 September 2021, to further compare patterns of post-vaccination AEs of adolescents with adults (aged 18 years and older).

### 2.2. Setting and Study Population

The incidence of AEs in adolescents and adults was compared after vaccination with BNT162b2 (Pfizer-BioNTech), as reported in the VAERS data. The inclusion criteria for adolescents were date (10 May 2021 to 30 September 2021), age (12–15 years), administration of the BNT162b2 vaccine, and CDC reporting total administration, while the inclusion criteria for adults was date (14 December 2020, to 30 September 2021), age (18 years and older), administration of the BNT162b2 vaccine, and CDC reporting total administration. The exclusion criterion for all datasets was an onset of > 84 days (number of days > 84). To ensure data integrity, we removed duplicates and extracted reports of individuals who experienced at least one AE after COVID-19 vaccination.

### 2.3. Outcomes

**Figure 2:**
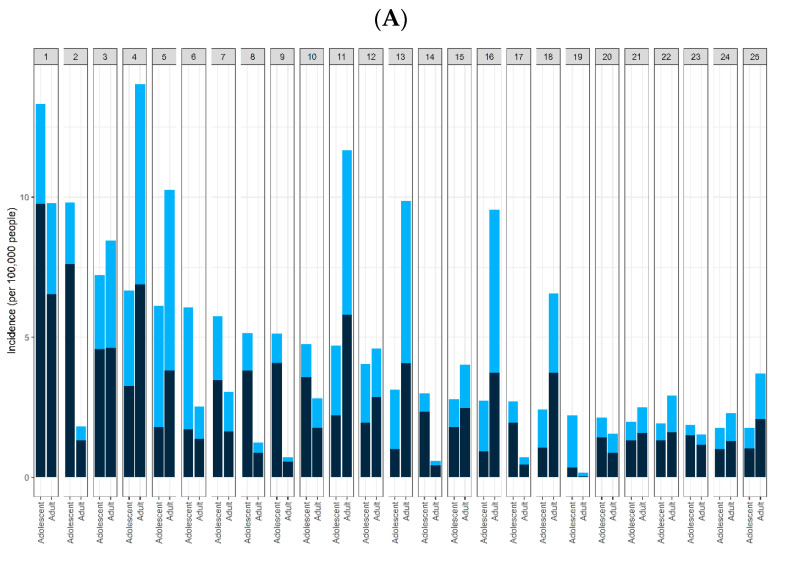

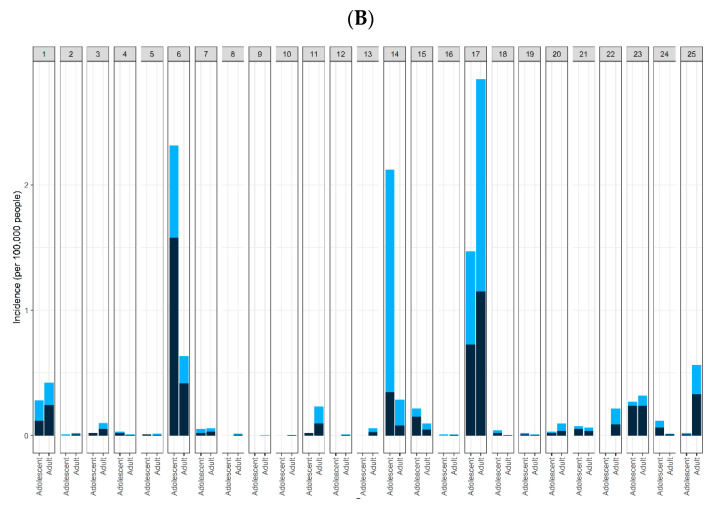
Incidence (per 100,000 people) of specific adverse events (AEs) among recipients of BNT162b2. (**A**) Incidence of the 25 common AEs after vaccination (1: dizziness; 2: syncope; 3: nausea; 4: headache; 5: pyrexia; 6: chest pain; 7: vomiting; 8: loss of consciousness; 9: pallor; 10: hyperhidrosis; 11: fatigue; 12: dyspnea; 13: pain; 14: unresponsive to stimuli; 15: rash; 16: chills; 17: fall; 18: pain in extremity; 19: troponin increased; 20: tremor; 21: urticaria; 22: asthenia; 23: flushing; 24: malaise; 25: injection site pain). The ranked top-25 common AEs are listed in order of incidence. (**B**) Incidence of 25 severe AEs in the 2 groups after vaccination (1: Bell’s palsy; 2: stroke, hemorrhagic; 3: stroke, ischemic; 4: encephalitis/myelitis/encephalomyelitis; 5: cerebral venous sinus thrombosis; 6: convulsions/seizures; 7: Guillain–Barré syndrome; 8: transverse myelitis; 9: acute disseminated encephalomyelitis; 10: narcolepsy/cataplexy; 11: pulmonary embolism; 12: acute respiratory distress syndrome; 13: acute myocardial infarction; 14: myocarditis/pericarditis; 15: appendicitis; 16: anemia; 17: lymphadenopathy; 18: lymphopenia; 19: neutropenia; 20: other thrombosis; 21: thrombocytopenia; 22: deep vein thrombosis; 23: anaphylaxis; 24: multisystem inflammatory syndrome in children/adults; 25: death). Dose 1 (first dose): black; dose 2 (second dose): light blue. This graph consists of data in which doses are recorded as 1 or 2.

We selected 25 severe AEs, including death, based on the recommendations of a focus group of three clinical experts. We also referenced two prior studies to identify the severe AEs considered in this study [17,18], listed in Table S1. In addition, we selected the top 25 common AEs, followed by the frequency of AEs, except for the 25 severe AEs (Figure 2).

### 2.4. Statistical Analysis

The obtained data were subjected to normality testing. Multiple logistic regression analysis was performed to determine the risk of AEs due to COVID-19 vaccination after adjusting for age, gender, onset days, and dose series (1st or 2nd dose). All statistics were two-tailed, and any *p* values < 0.05 were considered significant. We processed and analyzed the data using Python version 3.7.6 (Python Software Foundation, Delaware, United States) and R version 4.0.5 (R Foundation for Statistical Computing, Vienna, Austria).

## 3. Results

### 3.1. Population Characteristics

The characteristics of the adolescent population recorded in the VAERS database are shown in Table 1. From 10 May 2021, through 30 September 2021, 6304 individuals (mean age 13.6 ± 1.1 years, male: 47.3%, female: 52.7%) were reported to have experienced AEs after COVID-19 vaccination. The total administration in adolescents and adults was 9,252,431 and 226,033,301, respectively. The number of reported AEs in the administered BNT162b2 (Pfizer-BioNTech) vaccine was 6304 (68.14 per 100,000 people) and 162,993 (72.11 per 100,000 people) in adolescents and adults, respectively. The data were processed using a workflow process. The data from VAERS were collected from an adult population (18 years and over) and an adolescent population (12–15 years) vaccinated with BNT162b2 during the study period (Figure 1). Adolescents who completed the second dose accounted for 38% of the 6304 AEs reported.

### 3.2. Common AEs

The total reported incidence of common AEs was 10,099 cases (109.15 per 100,000 people). The incidence of severe AEs per 100,000 people among the recipients of the vaccines is shown in Figure 2A. Common AEs consisted of 9766 symptoms, excluding 25 severe AEs (138 symptoms). The top 25 most common AEs associated with vaccines administered to adolescents and adults are shown in Figure 2A. The five most frequent AEs in patients following vaccine administration were dizziness, syncope, nausea, headache, and pyrexia. In particular, the most common AEs with high incidence in adolescents were different from those in adults. Among the 25 most common AEs, 11 were specifically found in adolescents: syncope, chest pain, loss of consciousness, pallor, unresponsiveness to stimuli, falls, increased troponin, tremors, urticaria, flushing, and malaise. The incidence of pyrexia, chest pain, and troponin levels were higher after the second dose than the first dose (Figure 2A, Table S2).

### 3.3. Severe AEs

The total number of severe AEs reported was 634. The incidence of severe AEs was 6.85 per 100,000 vaccinated individuals (Figure 2B). The risk of myocarditis or pericarditis among adolescents was significantly higher in men than in women (odds ratio (OR) = 6.61, 95% confidence interval (CI) = 4.43 to 9.88), with a higher frequency noted after the second dose of the vaccine (OR = 8.52, 95% CI = 5.79 to 12.54).

With regards to lymphadenopathy, the OR of 1.96 (95% CI = 1.38 to 2.80) for males and 1.54 (95% CI = 1.09 to 2.17) for the second dose were significantly higher than those for females and first-dose vaccinations (Table 2). The incidence of myocarditis or pericarditis was higher after the second dose than after the first dose (Figure 2B). The risk of death among adolescents was significantly higher in terms of onset of symptoms (number of days) (OR = 1.08, 95% CI = 1.02 to 1.14) (Table 2).

For the comparison between adolescents and adults, the incidence (per 100,000 people) of the 25 severe AEs and the onset (days after vaccination) are shown in Table 3. MIS in children or adults was the most severe AE. Among all AEs, the relative risk (RR = 244.3, 95% CI = 31.27 to 1908.38) of MIS was highest compared to that in adults (Figure 2B, Table 3). In addition, some RR values were significantly high in adolescents compared to adults, including lymphopenia (RR = 97.72, 95% CI = 10.92 to 874.28), encephalitis/myelitis/encephalomyelitis (RR = 24.43, 95% CI = 3.44 to 173.43), myocarditis/pericarditis (RR = 19.60, 95% CI = 16.35 to 23.49), convulsions/seizures (RR = 5.70, 95% CI = 4.88 to 6.66), lymphadenopathy (RR = 4.58, 95% CI = 3.81 to 5.50), appendicitis (RR = 3.42, 95% CI = 2.14 to 5.46), Guillain–Barré syndrome (RR = 2.77, 95% CI = 1.19 to 6.43), and thrombocytopenia (RR = 2.28, 95% CI = 1.05 to 4.95) (Figure 2B, Table 3).

The most frequently reported severe AE after vaccination in adults was lymphadenopathy. In contrast, the incidence of myocarditis/pericarditis was 2.28 per 100,000 people in adolescents (Table 3). In addition, the incidence of convulsions/seizures in adolescents was 5.76 times higher than in adults (Table 3). The relative risk of pulmonary embolism (RR = 0.21, CI = 0.05–0.83) and death (RR = 0.07, CI = 0.02–0.27) was lower in adolescents than in adults. The incidence of deep vein thrombosis was significantly lower at 0 (Table 3).

## 4. Discussion

This study is the first to systemically analyze severe and common AEs following the administration of COVID-19 vaccines in adolescents using the VAERS data. Among adolescents, gender and dose series were associated with myocarditis/pericarditis and lymphadenopathies. Furthermore, the incidences of MIS, pulmonary embolism, deep vein thrombosis, and death were significantly higher in adolescents than in adults.

Recently, myocarditis and pericarditis, especially in adolescent males, have been reported as severe AEs of the COVID-19 vaccination [19,20,21,22,23,24,25,26] and COVID-19 infection [21]. Myocarditis has been proposed as a possible cardiac complication of COVID-19 as a result of infection with myocardial viruses such as parvovirus B19, human herpes virus, and Coxsackie virus which cause an inflammatory response in the host [27,28]. Indeed, many studies have suggested that the hyperinflammatory condition that can occur in patients with COVID-19 contributes to increased myocardial damage and mortality [29,30,31]. Similarly, a lower incidence of myocarditis has been reported in individuals after COVID-19 vaccination (0.3–5.0 per 100,000 vaccinated people) compared to after SARS-CoV-2 infection (1000–4000 per 100,000 people) [32]. According to Israel’s Ministry of Health, there were 148 cases of myocarditis within 30 days of vaccination among 10.4 million vaccinated individuals. The prevalence of myocarditis was five times higher in the 16–30-year-old group (1/20,000) compared to that in the general population (1/100,000) vaccinated with the same vaccine [33,34]. In Danish adolescents, the incidence of myopericarditis after BNT162b2 vaccination was revealed as 97 males and 16 females per million among individuals 12–17 years of age [35]. The present study similarly found that the risk of myocarditis following the BNT162b2 vaccination among adolescents (12–17 years old) was significantly higher in males than in females. However, the mechanisms by which mRNA vaccines induce myocarditis are not well understood. It may be associated with the SARS-CoV-2 virus-induced antagonism of cardiomyocytes or autoimmune antibodies produced by the host to induce a hypersensitivity inflammatory response [36]. The results of this study support previous findings that myocarditis/pericarditis is the most frequently reported severe AE after COVID-19 vaccination in adolescents [21,36,37]. The COVID-19 vaccine or SARS-CoV-2 infection increases the blood viscosity [12,38] and increases the expression levels of angiotensin-converting enzyme 2 [29]. As a result, underlying cardiac pathologies may be exacerbated, leading to myocarditis or pericarditis [12,39]. In previous studies, myocarditis and pericarditis were more frequent in men than in women [23,24,25] after the second dose of the vaccine [3]. Overall, the results of this study are similar to those of previous studies. Additionally, among the common AEs, pyrexia, chest pain, and troponin, which may be associated with myocarditis and pericarditis, increased in adolescents. These symptoms may be related to cardiac pathology. Therefore, these findings might have implications for the close monitoring of myocarditis and pericarditis in adolescents after COVID-19 vaccination.

MIS are more strongly associated with the COVID-19 virus [13] and vaccination [40] in adolescents than in adults. In addition, after BNT162b2 COVID-19 vaccination, MIS-C was described globally in several case reports. In a Danish study, after a second dose of the vaccine, MIS-C was reported in a 17-year-old with an onset of 5 days [41]. In a Turkish study, a 12-year-old had an onset of 27 days after the first dose of BNT162b2 [42]. Although the pathogenesis of COVID-19-associated MIS has not yet been elucidated, possible mechanisms of inflammatory processes were suggested by Jiang 2020: (1) antibodies to COVID-19 might exacerbate the disease by increasing viral entry into cells, (2) antibodies or T cells mediate cell damage or activation of inflammation, or (3) antibodies or T cells attack host antigens that cross-react or attack cells expressing viral antigens or mimic viral antigens [43].

In this study, the incidence and relative risk of MIS were significantly higher in adolescents than in adults. In fact, in the Chouchana study [44], 65 out of 159 children diagnosed with MIS in recent months after SARS-CoV-2 infection had severe neurological manifestations. These symptoms included direct neuro-invasion and an exaggerated immune response, which may be the most critical factors triggering MIS [45]. In addition, the RR values for lymphopenia, encephalitis/myelitis/encephalomyelitis, myocarditis/pericarditis, convulsions/seizures, lymphadenopathy, appendicitis, Guillain–Barré syndrome, and thrombocytopenia, which are symptoms associated with MIS, were also higher (*p* < 0.05) in adolescents than in adults.

Additionally, MIS may be accompanied by prominent gastrointestinal symptoms [46], a common feature of this syndrome in children, and can overlap with infectious or inflammatory abdominal conditions [47]. Furthermore, MIS may be accompanied by syncope, increased troponin levels, thrombocytopenia, lymphopenia, abnormal echocardiography findings (myocarditis, valvulitis, pericardial effusion, and coronary artery dilatation), abnormal abdominal ultrasonography findings (lymphadenopathy), and anemia [15]. Our results are thus consistent with those of previous studies.

### Limitations

The results of this study, compiled from a large collection of real-world data, show patterns similar to those in previous reports. Most VAERS data are voluntarily self-reported, indicating the potential for recall bias. In addition, this study did not control for all covariates because of limited information available on the VAERS data. Future studies should address the relationship between COVID-19 vaccines and individual biological characteristics, especially how underlying diseases and medications affect AEs after vaccination.

## 5. Conclusions

As a result of systemically analyzing severe and common AEs following the administration of the COVID-19 vaccine, the risk of the inflammatory response to COVID-19 vaccines such as myocarditis or pericarditis and MIS was significantly higher in adolescent males than in adults. The inflammation-related AEs may require close monitoring and management when vaccinating adolescents against COVID-19.

## Figures and Tables

**Table 1:** Demographic characteristics of individual cases with specific AEs in adolescent recipients of BNT162b2 vaccine (Pfizer-BioNTech).

	Severe AEs		
	Total	Dose1	Dose2
Female			
12	738	457	281
13	786	504	282
14	853	552	301
15	945	604	341
Sum	3322	2117	1205
Male			
12	714	407	307
13	668	403	265
14	763	463	300
15	837	497	340
Sum	2982	1770	1212
Total	6304	3887	2417

**Table 2:** Multiple regression analysis for major AEs by sex (coding male as 1 and female as 0), age (years), symptom onset (number of days), and dose series of vaccine (coding the 1st dose as 0 and the second dose as 1) as covariates. Dependent variables: incidence, independent variables: sex (coding male as 1 and female as 0), age (years), symptom onset (number of days), and dose series of vaccine.

	Lymphadenopathy			Myocarditis/Pericarditis		
	OR *	95% CI	*p*	OR *	95% CI	*p*
Sex (M/F)	1.96	1.38–2.80	<0.001	6.61	4.43–9.88	<0.001
Age (years)	0.91	0.78–1.06	0.23	1.53	1.33–1.76	<0.001
Symptom onset (days)	1.03	1.01–1.05	0.002	1.02	0.999–1.04	0.06
Dose series ^†^	1.54	1.09–2.17	0.01	8.52	5.79–12.54	<0.001
	**Multisystem inflammatory** **syndrome in children/adults**			**Death**		
	OR *	**95% CI**	***p***	OR *	**95% CI**	***p***
Sex (M/F)	0.91	0.28–2.99	0.88	NA	0–Inf	0.99
Age (years)	0.78	0.46–1.33	0.36	1.39	0.35–5.42	0.64
Symptom onset (days)	1.03	0.97–1.09	0.41	1.08	1.02–1.14	0.005
Dose series ^†^	1.27	0.38–4.22	0.70	1.08	0.06–18.39	0.96

* The odds ratio was calculated by multiple logistic regression analysis for each severe adverse event after adjusting for sex (reference: female), age, symptom onset (days), and dose series of vaccine as covariates. ^†^ Dose series: the 1st or 2nd dose.

**Table 3:** Incidence (per 100,000 people) of 25 severe AEs with onset days and relative risk after vaccination.

	Adolescents		Adults			
Symptom	Number	Incidence † of Events (Onset Day-Median)	Number	Incidence † of Events (Onset Day-Median)	Relative Risk * (95% CI)	Fisher’s Exact Test *p*
Bell’s palsy	26	0.28 (7)	728	0.32 (6)	0.87 (0.59–1.29)	0.57
Stroke, hemorrhagic	1	0.01 (51)	22	0.01 (6.5)	1.11 (0.15–8.24)	0.60
Stroke, ischemic	2	0.02 (12.5)	105	0.05 (5)	0.47 (0.11–1.89)	0.45
Encephalitis/myelitis/encephalomyelitis	2	0.02 (8.5)	2	0 (5.5)	24.43 (3.44–173.43)	0.01 *
Cerebral venous sinus thrombosis	1	0.01 (19)	16	0.01 (10.5)	1.53 (0.2–11.51)	0.49
Convulsions/seizures	197	2.13 (0)	844	0.37 (0)	5.7 (4.88–6.66)	<0.001 §
Guillain–Barré syndrome	6	0.06 (17)	53	0.02 (5)	2.77 (1.19–6.43)	0.03 ‡
Transverse myelitis	0	0	13	0.01 (8)	_	1.00
Acute disseminated encephalomyelitis	0	0	1	0 (8)	_	1.00
Narcolepsy/cataplexy	0	0	6	0 (1)	_	1.00
Pulmonary embolism	2	0.02 (18)	237	0.10 (11)	0.21 (0.05–0.83)	0.01 ‡
Acute respiratory distress syndrome	0	0	6	0 (13.5)	_	1.00
Acute myocardial infarction	0	0	59	0.03 (10)	_	0.18
Myocarditis/pericarditis	211	2.28 (2)	263	0.12 (3)	19.6 (16.35–23.49)	<0.001 §
Appendicitis	20	0.22 (2)	143	0.06 (6)	3.42 (2.14–5.46)	<0.001 §
Anemia	1	0.01 (29)	9	0 (3)	2.71 (0.34–21.42)	0.33
Lymphadenopathy	136	1.47 (1)	726	0.32 (1)	4.58 (3.81–5.5)	<0.001 §
Lymphopenia	4	0.04 (17)	1	0 (9)	97.72 (10.92–874.28)	<0.001 §
Neutropenia	2	0.02 (13)	8	0 (13.5)	6.11 (1.3–28.76)	0.06
Other thrombosis	3	0.03 (5)	107	0.05 (9)	0.68 (0.22–2.16)	0.80
Thrombocytopenia	7	0.08 (13)	75	0.03 (10)	2.28 (1.05–4.95)	0.04 ‡
Deep vein thrombosis	0	0	285	0.13 (8)	_	<0.001 §
Anaphylaxis	1	0.01 (0)	29	0.01 (0)	0.84 (0.11–6.18)	1.00
Multisystem inflammatory syndrome in children/adults	10	0.11 (1)	1	0 (1)	244.3 (31.27–1908.38)	<0.001 §
Death	2	0.02 (20)	726	0.32 (5)	0.07 (0.02–0.27)	<0.001 §

* The relative risk was calculated as ratio between two incidence proportions. † According to the CDC COVID-19 tracker, BNT162b2 (Pfizer-BioNTech) was administered to 9,252,431 individuals in adolescence (from 10 May 2021 to 30 September 2021) and was administered to 226,033,301 adults (aged 18 years and older, from 14 December 2020 to 30 September 2021), respectively. ‡ *p* < 0.05; § *p* < 0.001. In the case of transverse myelitis, acute disseminated encephalomyelitis, narcolepsy/cataplexy, acute respiratory distress syndrome, acute myocardial infarction, and deep vein thrombosis, the frequency of occurrence of adolescents was zero counts. Therefore, the results of relative risk regression were not presented in the table.

## Data Availability

All data analyzed in this study are public data from the Vaccine Adverse Event Reporting System (VAERS). VAERS is co-administered by the Centers for Disease Control and Prevention (CDC) and the U.S. Food and Drug Administration (FDA): https://vaers.hhs.gov/data/datasets.html (accessed on 11 April 2022).

## Supplementary Materials

The following supporting information can be downloaded at: https://www.mdpi.com/article/10.3390/vaccines10050744/s1, Table S1: Classification of severe AEs; Table S2: Multiple regression analysis for common AEs by sex (reference: female), age (years), symptom onset (number of days), and dose series of vaccine (coding the first dose as 0 and the second dose as 1) as covariates. Dependent variables: incidence, independent variables: sex (coding male as 1 and female as 0), age (years), symptom onset (number of days), and dose series of vaccine.
