# Supplementary material for: Adverse Events and Safety Profile of the COVID-19 Vaccines in Adolescents: Safety Monitoring for Adverse Events Using Real-World Data
Source: Vaccines (Basel). 2022 May 9;10(5):744. doi: 10.3390/vaccines10050744 (PMC9143867; doi:10.3390/vaccines10050744)
Supplement: Supplementary file 1 [file vaccines-10-00744-s001.zip › vaccines-1701832-supplementary.pdf]

Supplementary File

Table S1. Classification of severe AEs.

Table S2. Multiple regression analysis for common AEs by sex (reference: female), age (years), symptom onset (number of days), and dose series of vaccine (coding the first dose as 0 and the second dose as 1) as covariates. Dependent variables: incidence, independent variables: sex (coding male as 1 and female as 0), age (years), symptom onset (number of days), and dose series of vaccine.

Table S1. Classification of severe AEs.

Category	Severe AEs
Central nervous disorders	Bell's palsy
	Stroke, ischemia
	Stroke, hemorrhagic
	Encephalitis/myelitis/encephalomyelitis
	Cerebral venous sinus thrombosis
	Convulsion/seizures
	Guillain–Barré syndrome
	Transverse myelitis
	Acute disseminated encephalomyelitis
	Narcolepsy/cataplexy
Respiratory disorders	Pulmonary embolism
	Acute respiratory distress syndrome (ARDS)
Cardiac disorders	Acute myocardial infarction
	Myocarditis/pericarditis
Gastrointestinal disorders	Appendicitis
Hematologic disorders	Anemia
	Lymphadenopathy
	Lymphopenia
	Neutropenia
	Other thrombosis
	Thrombocytopenia
	Deep vein thrombosis
Others	Anaphylaxis
	Multisystem inflammatory syndrome in children/adults
	Death

Table S2. Multiple regression analysis for common AEs by sex (reference: female), age (years), symptom onset (number of days), and dose series of vaccine (coding the first dose as 0 and the second dose as 1) as covariates. Dependent variables: incidence, independent variables: sex (coding male as 1 and female as 0), age (years), symptom onset (number of days), and dose series of vaccine.

	Dizziness			Syncope			Nausea		
	OR	95% CI	*P*	OR	95% CI	*P*	OR	95% CI	*P*
Sex (M/F)	0.680	0.597 – 0.773	< 0.001	0.618	0.532– 0.717	< 0.001	0.755	0.641 – 0.890	0.001
Age (years)	1.184	1.118 – 1.254	< 0.001	1.119	1.049– 1.195	0.001	1.055	0.981 – 1.133	0.147
Onset (days)	0.872	0.841 – 0.905	< 0.001	0.678	0.618– 0.745	< 0.001	0.926	0.894 – 0.958	< 0.001
Dose series[a]	0.574	0.500 – 0.661	< 0.001	0.483	0.407– 0.572	< 0.001	0.981	0.829 – 1.160	0.820
	Headache			Pyrexia			Chest pain		
	OR	95% CI	*p*	OR	95% CI	*p*	OR	95% CI	*p*
Sex (M/F)	0.911	0.770 – 1.077	0.276	1.329	1.113 – 1.587	0.002	3.446	2.822– 4.207	< 0.001
Age (years)	0.927	0.861 – 0.998	0.045	0.898	0.831 – 0.971	0.007	1.254	1.155– 1.361	< 0.001
Onset (days)	1.000	0.986 – 1.014	0.968	1.012	1.000 – 1.025	0.047	1.020	1.008– 1.032	0.001
Dose series[a]	1.796	1.518 – 2.125	< 0.001	4.371	3.613 – 5.287	< 0.001	4.626	3.804– 5.626	< 0.001
	Loss of consciousness			Pallor			Unresponsive to stimuli		
	OR	95% CI	*p*	OR	95% CI	*p*	OR	95% CI	*p*
Sex (M/F)	0.853	0.705 – 1.033	0.104	3.593	0.675 – 0.993	0.042	0.640	0.497 – 0.824	0.001
Age (years)	1.283	1.176 – 1.400	< 0.001	1.037	1.022 – 1.212	0.014	1.137	1.019 – 1.269	0.022
Onset (days)	0.806	0.742 – 0.876	< 0.001	1.007	0.496 – 0.692	< 0.001	0.752	0.656 – 0.862	< 0.001
Dose series*	0.602	0.485 – 0.746	< 0.001	1.130	0.374 – 0.596	< 0.001	0.507	0.378 – 0.679	< 0.001
	Fall			Troponin increased			Tremor		
	OR	95% CI	*p*	OR	95% CI	*p*	OR	95% CI	*p*
Sex (M/F)	0.699	0.539 – 0.908	0.007	8.711	5.650 – 13.431	< 0.001	0.787	0.589 – 1.051	0.104
Age (years)	0.863	0.790 – 0.942	0.001	1.441	1.258 – 1.650	< 0.001	1.233	1.083 – 1.405	0.002
Onset (days)	1.104	0.985 – 1.238	0.088	1.022	1.005 – 1.040	0.010	0.967	0.926 – 1.010	0.128
Dose series*	0.686	0.518 – 0.910	0.009	8.558	5.847 – 12.524	< 0.001	0.843	0.623 – 1.140	0.268
	Urticaria			Flushing			Malaise		
	OR	95% CI	*p*	OR	95% CI	*p*	OR	95% CI	*p*
Sex (M/F)	1.171	0.873 – 1.572	0.291	0.869	0.638 – 1.184	0.373	0.967	0.708 – 1.321	0.832
Age (years)	0.851	0.747 – 0.970	0.016	1.134	0.988 – 1.302	0.075	1.077	0.937 – 1.237	0.297

Onset (days)	1.018	0.997 – 1.039	0.089	0.673	0.539 – 0.842	0.001	1.000	0.972 – 1.028	0.999
Dose series*	0.798	0.584 – 1.091	0.157	0.423	0.286 – 0.627	< 0.001	1.191	0.867 – 1.635	0.281

*Dose series: The 1st or 2nd dose

OR: Odds Ratio, 95% CI: 95% confidence intervals, *p*: *p*-values
